# 1255. Filipino Health Care Professionals’ Knowledge, Attitude and Perception regarding Drug-Susceptible and Drug-Resistant Tuberculosis in a High TB Burden City in Central Luzon: A Cross- Sectional Study

**DOI:** 10.1093/ofid/ofad500.1095

**Published:** 2023-11-27

**Authors:** J E R O M E G MANZANO, Divina Cristy Redondo-Samin

**Affiliations:** Dr. Paulino J. Garcia Memorial Research and Medical Center; Philippine General Hospital, Cabatuan, Isabela, Philippines; Dr. Paulino J. Garcia Memorial Research and Medical Center, Cabanatuan City, Nueva Ecija, Philippines

## Abstract

**Background:**

Tuberculosis (TB) is one of the major diseases responsible for the public health and economic crisis in low-income countries, with the Philippines as one of the eight countries in 2020 that accounted for two thirds of the new TB cases worldwide. Its three most populous regions which are the National Capital Region, Calabarzon and the Central Luzon Region reported the highest number of TB cases in 2015. One important consideration is that health care providers’ knowledge, attitude and perception regarding TB largely affects the success of TB treatment.

**Methods:**

This study assessed the knowledge, attitude and perception among health care professionals who manage tuberculosis, using a validated questionnaire regarding drug-susceptible and drug-resistant tuberculosis in Cabanatuan City, Nueva Ecija. Cross-sectional study was used in this research. All health care professionals assigned in each identified health facility were asked to participate in the study. After obtaining informed consent, a self-administered questionnaire was given to all participants to answer. Descriptive statistics and Chi-square tests were used in data analysis.

**Figure 1**

**Figure:**
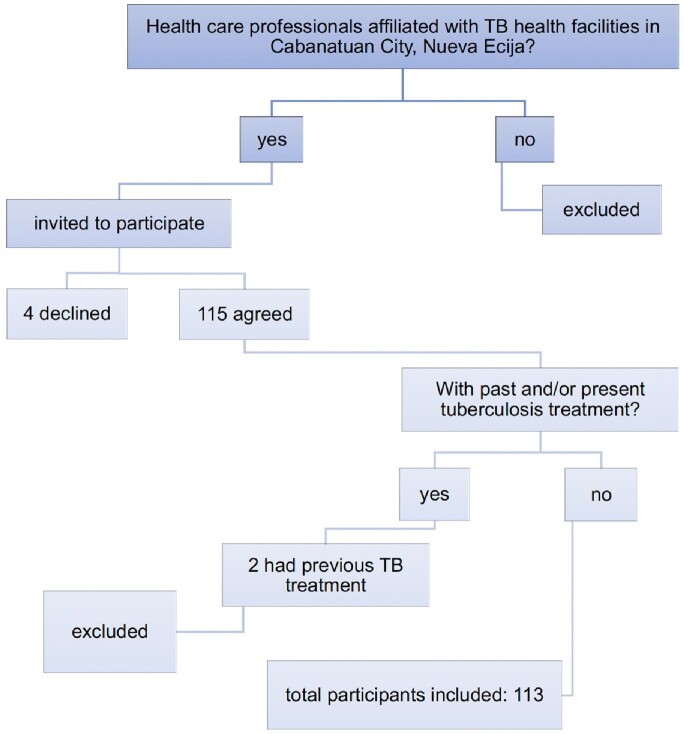

Selection of Study Participants through Purposive Sampling

**Results:**

1. A total of 113 participants were included in the study. The participants were all HCPs with most of them less than 30 years of age, and females.
2. In this study, nurses and physicians were found to have good knowledge about drug- susceptible tuberculosis. However, when HCP’s were asked about MDR-TB, physicians were found to account for the majority of participants who were able to obtain the correct response.
3. As with statistical variation between training on DOTS and their knowledge about tuberculosis, it was found that those who had training on DOTS were more knowledgeable about tuberculosis compared to those who had no training at all. The results were equally supported by other studies where they revealed that training is essential in enhancing the knowledge of HCP’s in treating and managing tuberculosis.

**Table 1**

**Figure:**
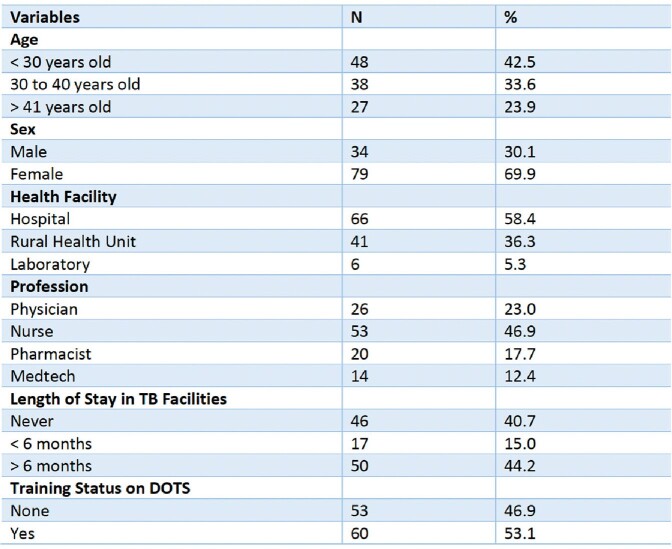

Socio-Demographic Profile of HCPs in Cabanatuan City Nueva Ecija

**Table 2**

**Figure:**
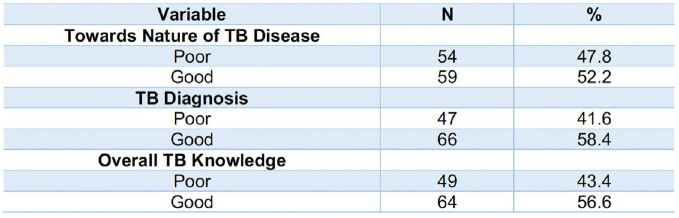

Knowledge of HCPs on Diagnosis and Nature of Tuberculosis

**Table 3**

**Figure:**
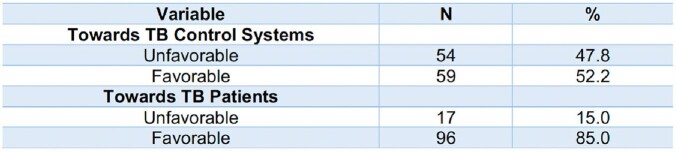

Attitude of HCPs towards Patients with Tuberculosis and the TB Control Systems

**Conclusion:**

The lack of training may have largely contributed to the poor knowledge of HCPs which may possibly hinder the success of providing TB treatment. It is therefore a paramount consideration that prior to the HCP’s assignment in TB DOTS centers, all HCPs must first undergo training in order to manage TB treatment properly and successfully.

**Table 4**

**Figure:**
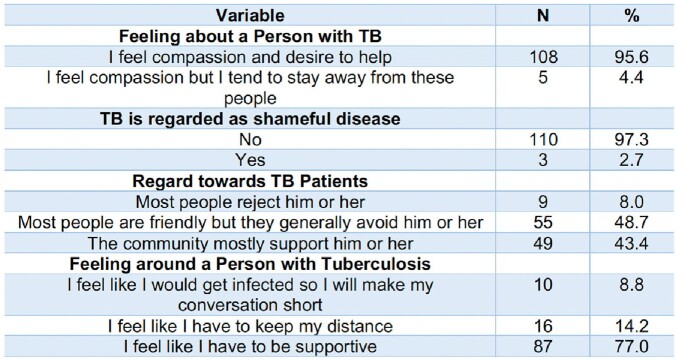

Perception of HCPs towards Tuberculosis

**Table 5.1**

**Figure:**
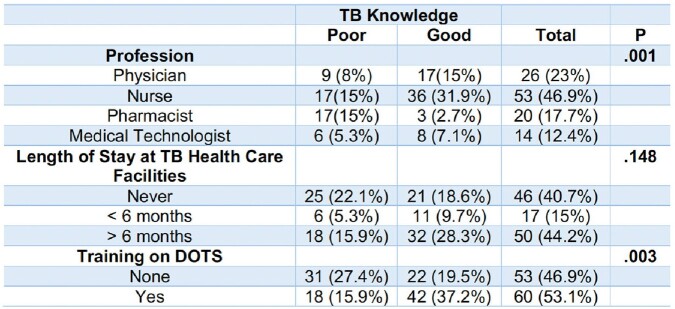

Significant Difference between overall TB Knowledge of HCPs and the Socio-Demographic Profile

**Table 5.2**

**Figure:**
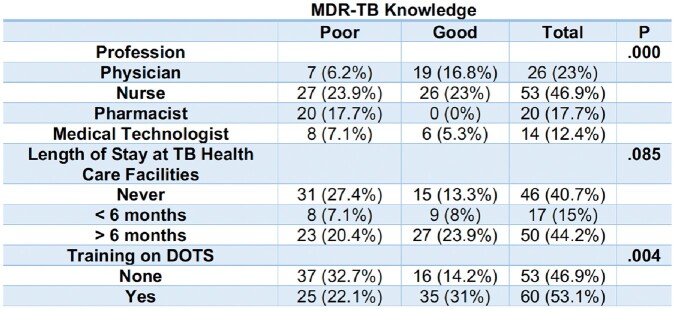

Significant Difference between MDR-TB Knowledge of HCPs and the Socio-Demographic Profile

**Disclosures:**

**All Authors**: No reported disclosures

